# Retraction Note: Indoleamine 2,3-Dioxygenase 1 (IDO1) Promotes Cardiac Hypertrophy via a PI3K-AKT-mTOR-Dependent Mechanism

**DOI:** 10.1007/s12012-023-09779-5

**Published:** 2023-01-24

**Authors:** Yang Liu, Shuang Li, Zhanqun Gao, Shuangjia Li, Qingyun Tan, Yanmei Li, Dongwei Wang, Qingdong Wang

**Affiliations:** 1 Emergency Department, First Affiliated Hospital of Jiamusi University, Jiamusi, China; 2 Department of Anesthesiology, First Affiliated Hospital of Jiamusi University, Jiamusi, China

**Retraction Note to: Cardiovascular Toxicology (2021) 21:655–668** 10.1007/s12012-021-09657-y

The authors have retracted this article because after publication they found that they were unable to replicate the key results from the cardiomyocyte size experiments. It was also noted that images in Fig. 4B and E had been used in previous publications [1, 2]. Therefore the authors no longer have confidence in the reliability of the results presented in this article. All authors agree to this retraction.
